# Celebrating the 40th anniversary of Health Policy and Planning

**DOI:** 10.1093/heapol/czaf097

**Published:** 2026-01-23

**Authors:** Anne Mills, Gill Walt, Lucy Gilson, Virginia Wiseman

**Affiliations:** Department of Global Health and Development, London School of Hygiene and Tropical Medicine, 15-17 Tavistock Pl, London WC1H 9SH, United Kingdom; Department of Global Health and Development, London School of Hygiene and Tropical Medicine, 15-17 Tavistock Pl, London WC1H 9SH, United Kingdom; Health Policy and Systems Division, School of Public Health, University of Cape Town, Anzio Rd, Observatory, 7925 Cape Town, South Africa; Department of Global Health and Development, London School of Hygiene and Tropical Medicine, 15-17 Tavistock Pl, London WC1H 7SH, United Kingdom; Department of Global Health and Development, London School of Hygiene & Tropical Medicine, 15-17 Tavistock Pl, London WC1H 9SH, United Kingdom; Kirby Institute, University of New South Wales, Wallace Wurth Building, Cnr High St & Botany St, Kensington, NSW 2033, Australia

## Introduction

This year, 2026, we celebrate the 40th anniversary of this Journal, ‘Health Policy and Planning’. The journal was conceived in 1986 by Patrick Vaughan, Professor of Epidemiology at the London School of Hygiene and Tropical Medicine (LSHTM). We sadly marked his recent death in Issue 8 of 2025. He and Gill Walt, now Emeritus Professor of International Health Policy, were its founding editors; Anne Mills was co-editor in 1986/7 and 1993–2001 and remained associated with the journal for a further 20 years. We are joined by the two current Editors-in-Chief in this reflection on the evolution of Health Policy and Planning (HPP) over the last 40 years.

### The purpose and content of the journal, then and now

The original purpose of the journal, as stated in ‘To Our Readers’ (https://academic.oup.com/heapol/article/1/1/3/585405) in the first issue, was to create an international forum in which issues in health policy, planning, management, and evaluation in relation to what was then termed the ‘developing’ world could be openly discussed. Its intended readership included those working in health services and in governments, universities, and international agencies. Four key emphases were listed:

- A multi-disciplinary approach to health—encompassing, e.g. geography, economics, health planning, demography, epidemiology and statistics
- An international perspective—with the expressed desire that most of the material will be written by authors living in developing countries
- Current issues and debates—especially provocative articles that explore, expose and question conventional wisdom
- A balance between theoretical and practical approaches

HPP remains true to this original purpose, if updated to contemporary terminology. The word ‘planning’ has disappeared, along with ‘developing countries’, and ‘health systems’ has been added; the current focus is worded as ‘design, implementation and evaluation of health policies and health systems in low- and middle-income countries’. Policymakers, public health researchers and practitioners remain its targeted readership. Perhaps the most notable change has been the journal’s strap line—from ‘A journal on health in development’ to ‘The journal on health policy and systems research’. In 1986, the term ‘health systems’ was not in general use, and health policy and systems research was yet to be defined as a distinct sub field of health research. Rather, it was considered important then to root the journal in a development focus, emphasizing the links between health and other sectors.

The core of HPP has always been original articles, both empirical research and literature reviews. Given the concern of the journal to be relevant to policy and practice and to support researchers and decision makers in the health system and policy arenas, various other types of material have been solicited over time. The journal’s ‘How to do (or not to do)’ series, introduced in 2009, is very popular and consistently highly cited and downloaded. This series clearly meets the demands of readers for practical advice on how to use a particular research or analytical method. The most long-lasting series, which has survived the test of time, has been ‘10 best resources’ (originally labelled ‘books’), present in volume 1 issue 1 and still continuing to this day. These communications identify and outline the 10 most useful resources from a range of sources to help facilitate a better understanding of a particular issue in global health.

Over the decades HPP has expanded, reflecting more general contextual influences, such as the growth in volume of academic research across the world and greatly increased volume of development assistance for health, and more specifically, greater interest in the applied areas of health policy and systems research. Between 1986 and 2003, four issues a year were produced. This was then increased in 2004 to 6 issues a year, to 8 in 2012, and to 10 from 2015 up to the present day. This has resulted in a total of 250 issues over HPP’s 40 years.

In 2001 supplements were introduced, providing the opportunity to present a peer-reviewed collection of original articles on a common theme. Over the past 10 years, there has been an average of three to five supplements a year. A total of 37 supplements have been published since 2001—a major and valuable addition to the material in the core issues. Supplements have also been produced alongside major conferences and meetings including the Global Symposium on Health Systems Research. In 2018, HPP joined with the Alliance for Health Policy and Systems Research and Health Systems Global to launch their new Women’s Publication Mentorship Programme. To date, HPP has published two supplements with 17 articles led by first-time women authors and their mentors supported through this programme.

In 2024, 105 countries were named in the addresses of authors submitting to the journal, of which 67% were low- and middle-income countries (LMIC). Authors of published articles came from 53 countries, of which 76% were LMIC, suggesting that the journal has been able to deliver on its aspiration to greatly increase the share of material written by authors living in these countries. The 20 countries with the greatest number of submitting authors, and top 20 countries for published authors, are presented in Table 1. Sixty-five percent of the top 20 countries for authors of submitted articles were LMICs, and 60% for published articles. Of the LMICs in these tables, only India, South Africa and Brazil were included in the first volume of 1986, indicating the huge growth in LMIC health policy and systems research activity over the life of the journal. Nonetheless, total numbers of submitting and published authors from high- income countries still greatly exceed those from LMICs, especially for published articles, suggesting a clear unfinished agenda in ensuring LMICs and their researchers are fully represented in the journal’s published output.

**Table 1.** Top 20 countries as reflected in address of submitting and published authors, 2024.

Authors in submitted articles		Authors in published articles	
Country named in author addresses	Number of authors per country	Country named in author addresses	Number of authors per country
China	160	United Kingdom	35
United States	86	China	16
United Kingdom	83	Australia	20
Australia	53	United States	20
Ethiopia	23	India	11
India	29	South Africa	10
South Africa	24	Germany	3
Italy	14	Netherlands	3
Nigeria	19	Nigeria	6
Iran, Islamic Republic of	11	Canada	3
Canada	22	Norway	3
Netherlands	12	Switzerland	9
Malaysia	11	Ghana	4
Turkey	10	Burkina Faso	3
Brazil	9	Kenya	3
Kenya	19	Malawi	3
Taiwan, Province of China	8	Uganda	3
Uganda	14	Argentina	2
Colombia	7	Mexico	2
Malawi	9	Philippines	2

Technology has transformed the publishing industry over the last 40 years. Up until 2024 the journal was available in hard copy only. Now there is world-wide access to the online editions, with nearly 2 m downloads of articles in 2024. The entire corpus of published material from 1986 onwards was put online in 2024.

Downloads and citations provide an indication of the interests of readers. Table 2 lists the 10 most downloaded articles, and Table 3 the most frequently cited articles. Policy analysis and health economics-related topics dominate both these lists, along with an emphasis on articles which offer methodological guidance, practical advice and evidence synthesis. The popularity of policy analysis and health economics may well reflect the journal’s positioning in the highly applied research space, and the dedicated sections for such articles.

**Table 2.** Top downloaded articles of all time (as at 05/09/2025).

Title	Authors	Year published	Number of full-text downloads	DOI
Stakeholder analysis: a review	Brugha R, Varvasovszky Z	2000	137 091	10.1093/heapol/15.3.239
A stakeholder analysis	Varvasovszky Z, Brugha R	2000	90 134	10.1093/heapol/15.3.338
Document analysis in health policy research: The READ approach	Dalglish SL, Khalid H, McMahon SA	2000	62 799	10.1093/heapol/czaa064
'Doing’ health policy analysis: methodological and conceptual reflections and challenges	Walt G, Shiffman J, Schneider H, Murray SF, Brugha R, Gilson L	2008	41 973	10.1093/heapol/czn024
Reforming the health sector in developing countries: the central role of policy analysis	Walt G, Gilson L	1994	41 601	10.1093/heapol/9.4.353
Constructing socio-economic status indices: how to use principal components analysis	Vyas S, Kumaranayake L	2006	34 043	10.1093/heapol/czl029
How to do (or not to do) … Designing a discrete choice experiment for application in a low-income country	Mangham JL, Hanson K, McPake B	2009	30 528	10.1093/heapol/czn047
Calculating QALYs, comparing QALY and DALY calculations	Sassi F	2006	28 819	10.1093/heapol/czl018
Sin taxes and their effect on consumption, revenue generation and health improvement: a systematic literature review in Latin America	Miracolo A, Sophiea M, Mills M, Kanavos P	2021	28 311	10.1093/heapol/czaa168
Coca-Cola’s political and policy influence in Mexico: understanding the role of institutions, interests and divided society	Gómez EJ	2019	28 278	10.1093/heapol/czz063

**Table 3.** Top cited articles of all time (as at 05/09/2025).

Title	Authors	Year published	Citations	DOI
Constructing socio-economic status indices: how to use principal components analysis	Vyas S, Kumaranayake, L	2006	2336	10.1093/heapol/czl029
Reforming the health sector in developing-countries—the central role of policy analysis	Walt G, Gilson L	1994	828	10.1093/heapol/9.4.353
Measuring social capital within health surveys: key issues	Harpham T, Grant E, Thomas E	2002	679	10.1093/heapol/17.1.106
Stakeholder analysis: a review	Brugha R, Varvasovszky Z	2000	617	10.1093/heapol/15.3.239
Overcoming barriers to health service access: influencing the demand side	Ensor T, Cooper S	2004	581	10.1093/heapol/czh009
Doing health policy analysis: methodological and conceptual reflections and challenges	Walt G, Shiffman J, Schneider, H, Murray SF, Brugha R, Gilson L	2008	558	10.1093/heapol/czn024
Thirty years of national health insurance in South Korea: lessons for achieving universal health care coverage	Kwon S	2009	421	10.1093/heapol/czn037
A stakeholder analysis	Varvasovszky Z, Brugha R	2000	407	10.1093/heapol/15.3.338
How to do (or not to do) Designing a discrete choice experiment for application in a low-income country	Mangham L J, Hanson K, McPake B	2009	398	10.1093/heapol/czn047
Calculating QALYs, comparing QALY and DALY calculations	Sassi F	2006	360	10.1093/heapol/czl018

### Governance, management and requirements of the journal, then and now

In 1986 there were 2 editors, both UK-based, and 28 editorial advisors, 16 based in high income countries and 12 in LMICs. The role of editorial advisors was primarily to help with extending the reach of the journal by encouraging submissions. With the growth of the journal, the development of the multi-disciplinary field of health policy and systems research, and the need to ensure high quality in multiple areas, the management of the journal has changed hugely over the decades. There are still two editors, but now named editors-in-chief, overseeing a team of section editors. The journal now publishes in four subject sections, namely Health Economics, Health Policy Processes, Health Systems Research, and Implementation Research and Evaluation, with several editors responsible for managing submissions to each subject section. Half of the journal’s section editors are based in LMICs and there is at least one LMIC-based editor in each subject section. In addition, there are editors for statistics and supplements. The Editors-in-Chief both have dual appointments, between LSHTM and an external university, Cape Town and New South Wales, helping to extend the journal’s reach beyond LSHTM.

Over the last decade there has been a re-doubled effort to broaden the geographical coverage of the journal, including coverage of the poorest countries and those in transition, to encourage and support LMIC researchers to publish in HPP, and to address the power imbalance between LMIC and high-income country researchers. In 2023, the authorship policy was adopted that, as a general rule, for empirical research and research using secondary data, only articles that include at least one co-author based in one of the countries where the research was undertaken would be published. Author requirements were amended to include a statement reflecting on inclusivity within the authors’ group including balance in dimensions such as gender, seniority, and regional location.

In 2025, the editors-in-chief established a new look Editorial Advisory Board (EAB). New members were appointed from Africa, Latin America, and Asia to advise on the changing landscape of health policy and health systems research in these regions, and to explore how better to engage and support authors. In addition, the new EAB includes two highly experienced ‘super reviewers’ whose role is to help speed up the journal’s review process whilst maintaining the quality of reviews—an enduring challenge for many journals. The super reviewers either use their extensive networks to expand HPP’s pool of reviewers or undertake reviews in their areas of expertise. We will continue to closely review progress in geographical engagement and processing times in the years ahead.

Access to the journal has been of concern from the very early days, given the annual subscription needing to be paid to obtain the journal. In the 1986 ‘To our Readers’ preface, all that could be said was to alert readers to UNESCO coupons which enabled payment in local currency, and to potential sources of non-governmental and international support. Subsequent years saw the introduction of a free annual subscription for authors of the best papers in each year. With the global changes in the financial model of the journal publishing industry, HPP became a fully open access journal in 2024, meaning free world-wide access to articles, but requiring payment of article processing charges. Full and partial waivers are available for corresponding authors with a primary affiliation in a low- and middle-income country. Authors whose institutes have a Read and Publish agreement with OUP may have the article processing charge covered under these agreements. A 25% reduction on charges is also available to members of Health System Global, the global association of health policy, systems research and practice communities, and reviewers who have reviewed 2 or more articles in a calendar year receive, in the following year, a 15% discount on the charge for an accepted paper.

### The future

HPP will continue to emphasize the concerns that have driven the journal since its inception, as it looks to the future. Section editors and new EAB members will identify papers on topics of growing importance to LMIC health policy and systems, such as climate change, the role of Artificial Intelligence in health systems, rapid urbanization, the changing donor funding landscape, and health system preparedness to respond to varying disease shocks. At the same time, HPP will maintain its interest in understanding and responding to enduring systemic challenges such as strengthening primary care, health governance and health financing reform. It will also continue to publish papers from diverse policy areas that focus on analysing the process of policy change across all stages of the policy cycle, and that report implementation research and evaluation. Across topics and sections we will maintain our focus on high quality research and embrace new methodological approaches—from participatory and co-production research to critical, theory-based research and evaluation. Our core concern will continue to be supporting work that offers value for health system development in LMICs—by sharing experience, encouraging methodological reflection and stimulating policy debate.

As a journal, HPP is fortunate in being able to build on its worldwide community of readers, reviewers and authors, who have been fundamental to its success. It is also indebted to its excellent section editors, guest editors, and to past and present advisory board members. As founding editors and current editors-in-chief, we would like to acknowledge the intervening senior editors, who all have contributed to its growth and development—Kara Hanson, Richard Coker, Sara Bennett and Sandra Mounier-Jack.

## Ethical approval

Ethical approval for this type of study is not required by LSHTM.

## Data Availability

Data cannot be shared for commercial reasons.

